# Comparative Evaluation of the Angiogenic Potential of Hypoxia Preconditioned Blood-Derived Secretomes and Platelet-Rich Plasma: An In Vitro Analysis

**DOI:** 10.3390/biomedicines8010016

**Published:** 2020-01-16

**Authors:** Philipp Moog, Katharina Kirchhoff, Sanjar Bekeran, Anna-Theresa Bauer, Sarah von Isenburg, Ulf Dornseifer, Hans-Günther Machens, Arndt F. Schilling, Ektoras Hadjipanayi

**Affiliations:** 1 Experimental Plastic Surgery, Clinic for Plastic, Reconstructive and Hand Surgery, Klinikum Rechts der Isar, Technische Universität München, D-81675 Munich, Germany; philippmoog@web.de (P.M.); katharina.kirchhoff@icloud.com (K.K.); sanjar.bekeran@gmx.de (S.B.); anna.theresa.bauer@googlemail.com (A.-T.B.); dornseifer@ustransplant.de (U.D.); e.hadjipanayi@googlemail.com (E.H.); 2 Department of Plastic, Reconstructive, Hand and Burn Surgery, Bogenhausen Hospital, D-81925 Munich, Germany; dr.isenburg@neuhannlorenz-isenburg.com; 3 Department of Plastic, Reconstructive and Aesthetic Surgery, Isar Klinikum, D-80331 Munich, Germany; 4 Department of Trauma Surgery, Orthopedics and Plastic Surgery, Universitätsmedizin Göttingen, D-37075 Göttingen, Germany; arndt.schilling@med.uni-goettingen.de

**Keywords:** peripheral blood cells, blood-derived therapy, hypoxia, angiogenesis, platelet rich plasma (PRP), hypoxia preconditioned plasma, hypoxia preconditioned serum

## Abstract

Blood-derived factor preparations are being clinically employed as tools for promoting tissue repair and regeneration. Here we set out to characterize the in vitro angiogenic potential of two types of frequently used autologous blood-derived secretomes: platelet-rich plasma (PRP) and hypoxia preconditioned plasma (HPP)/serum (HPS). The concentration of key pro-angiogenic (VEGF) and anti-angiogenic (TSP-1, PF-4) protein factors in these secretomes was analyzed via ELISA, while their ability to induce microvessel formation and sprouting was examined in endothelial cell and aortic ring cultures, respectively. We found higher concentrations of VEGF in PRP and HPP/HPS compared to normal plasma and serum. This correlated with improved induction of microvessel formation by PRP and HPP/HPS. HPP had a significantly lower TSP-1 and PF-4 concentration than PRP and HPS. PRP and HPP/HPS appeared to induce similar levels of microvessel sprouting; however, the length of these sprouts was greater in HPP/HPS than in PRP cultures. A bell-shaped angiogenic response profile was observed with increasing HPP/HPS dilutions, with peak values significantly exceeding the PRP response. Our findings demonstrate that optimization of peripheral blood cell-derived angiogenic factor signalling through hypoxic preconditioning offers an improved alternative to simple platelet concentration and release of growth factors pre-stored in platelets.

## 1. Introduction

Over the past decade, there has been a growing interest in the therapeutic application of autologous blood-derived products for the treatment of various skin pathologies and chronic wounds [1,2,3,4,5]. Under physiological conditions, a wound can rapidly regenerate through a series of well-defined wound healing stages, namely haemostasis, inflammation, proliferation and angiogenesis, and eventually tissue remodelling [6,7]. The efficiency and reliability of this complex system of cellular responses, which are powered by a myriad of molecular cascades, suggests that targeted reproduction of the basic foundation underlying the natural process would provide a useful tool for promoting tissue repair and regeneration on-demand. These treatments could be readily applied in the clinical setting, despite an incomplete understanding of the precise set of rules that govern such mechanisms, at present. This rational has given rise to the clinical use of peripheral blood cell-derived growth factor mixtures, which in effect represent cumulative samples of the molecular output of the key cell types that participate in the wound healing program. During the haemostatic phase, platelets play a paramount role by concentrating at the site of injury, and releasing a plethora of growth factors through degranulation [8,9,10,11]. The clot fibrin matrix thereafter provides a scaffold for migrating peripheral blood cells (PBCs), e.g., neutrophils, macrophages, lymphocytes, and other cell types (e.g., fibroblasts, endothelial cells) [12,13,14]. As a result of vascular trauma and the consequent disruption in oxygen supply, PBCs are exposed to local hypoxia, which leads to the production and release of pro-angiogenic growth factors that stimulate new vessel formation and reestablishment of the wound bed’s microcirculation [15,16,17,18]. Thus, the main purpose of the application of blood-derived secretomes in wounded or ischaemic tissues is the targeted stimulation and support of the cellular responses that naturally drive angiogenesis and tissue repair, via protein growth factor signalling. The utilization of autologous growth factors provides a means of offering a personalised treatment to each individual patient, while autologous blood-derived products present unique advantages compared to allogeneic/xenogeneic therapies, by minimizing the risk of infection and adverse immunological reactions [2,5,16,19,20,21].

**Figure 1:**
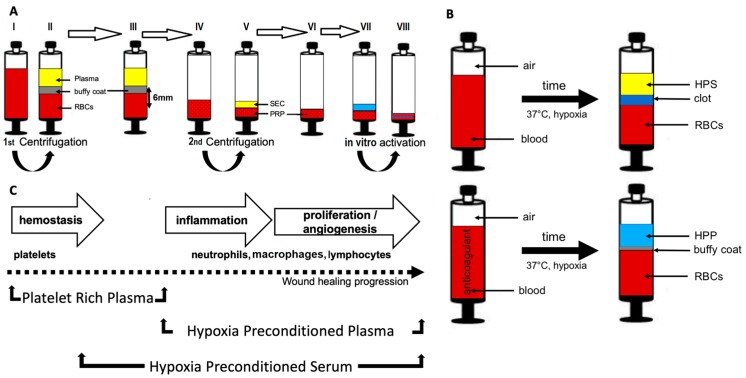
Method of preparation of different blood-derived secretomes and their correlation with the physiological wound healing phases. (**A**) Preparation of platelet-rich plasma (PRP). Main procedure steps are shown by arrows. (I) Tube with anticoagulant after blood sampling. (II) After 1st centrifugation, blood has been separated into the shown layers. (III) Collection of all content (including the buffy coat) above the mark (6 mm), and (IV) transfer to another tube without anticoagulant before re-centrifugation. (V) After 2nd centrifugation, there is a division into serum component (SEC) and PRP. (VI) Separation of PRP (0,5 mL) from the SEC. (VII) Activation of PRP with Thrombin and CaCl_2_, or by CaCl_2_ only. (VIII) Activated PRP as a final product (blue dots represent released growth factors in PRP). (**B**) Preparation of hypoxia preconditioned secretomes (HPS, HPP). Blood is allowed to clot (top), or anticoagulated (bottom), and PBCs (in clot or buffy coat) are preconditioned under pericellular (local) hypoxia (~1% O_2_) and physiological temperature (37 _o_C) for 4 to 7 days. Sedimentation passively separates growth factor-rich hypoxia preconditioned serum (HPS) or hypoxia preconditioned plasma (HPP) from clot or buffy coat, respectively, and red blood cells (RBCs) at the bottom (**C**) Overview of the correlation of the two categories of blood-derived secretomes with the key cell types involved in wound healing, and the corresponding wound healing phases. PRP comprises an increased concentration of platelet-derived growth factors, which is naturally achieved during hemostasis through platelet concentration within the fibrin clot, while HPS and HPP mostly comprise hypoxia-induced factors, which are naturally produced by PBCs during the inflammatory and proliferative phases.

Platelet-rich plasma (PRP), currently accepted as the gold-standard of blood-based regenerative therapies [3,4], is a firmly defined fraction of the plasma component of peripheral blood, with a platelet concentration significantly above the normal limit (normal human plasma platelet count: 150.000–450.000 platelets/μL) [22,23,24,25,26]. The aim of the PRP procedure is to achieve an increased concentration of platelet-derived protein growth factors by increasing the plasma concentration of platelets (Figure 1A). Typically, the concentration of platelets in PRP is about three to six times the normal platelet concentration in peripheral blood [27,28], and can therefore generate an elevated concentration of growth factors [11,27,29]. This supraphysiological factor concentration in PRP is achieved through platelet activation, degranulation and release of their stored growth factors [11,29,30,31], e.g., VEGF (vascular endothelial growth factor), PDGF (platelet-derived growth factor), EGF (epidermal growth factor), IGF-1 (insulin-like growth factor-1), bFGF (basic fibroblast growth factor), TGF-beta1 (transforming growth factor beta-1), TSP-1 (thrombospondin-1) and PF-4 (platelet factor-4) [23,31,32,33,34,35]. Evidently, PRP is a composite mixture of both angiogenesis-promoting (e.g., VEGF, PDGF) and angiogenesis-inhibiting (e.g., TSP-1, PF-4) protein factors, which makes the analysis of this secretome’s net angiogenic effect(s) inherently complex. Indeed, the functional utility of PRP as a regenerative agent has not yet been fully clarified, despite its almost 20 years of use [3,4]. At an in vitro level, PRP is known to stimulate many cell types involved in wound repair, such as dermal fibroblasts and endothelial cells [36,37,38,39]. The clinical data on the efficacy of PRP for skin regeneration and treatment of chronic wounds are so far inconclusive [35,40].

Vascularization of the wound bed is achieved through angiogenesis, which is required for adequate supply of nutrients/oxygen and maintenance of cell viability. Following platelet aggregation and haemostasis, hypoxia (i.e., low oxygen tension) acts as the strongest stimulus for angiogenic induction [17,18,41]. Utilization of hypoxia as a tool to stimulate angiogenesis on-demand harnesses the innate biological mechanism that naturally promotes new vessel formation in the body, in both physiological (e.g., embryogenesis) and pathological states (e.g., ischemia, wound healing, tumour formation) [16,17,18,42,43]. Hypoxia preconditioned blood-derived secretomes can be generated through the process of “extracorporeal wound simulation”, which entails peripheral blood incubation under physiological temperature (37 °C) and hypoxia (1–10% O_2_) (Figure 1B) [2,5,19,44]. As previously demonstrated, pericellular hypoxia can be achieved in situ within the blood incubation chamber, by adjusting the blood volume per unit area (BVUA), i.e., the PBC seeding density, and hence cellular O_2_ consumption, thus overcoming the need for an oxygen-controlling incubator [2,13,19]. It is already established that PBCs respond to stress (e.g., hypoxia, ischemia, inflammation, ultrasound) by upregulating a range of pro-angiogenic growth factors such as VEGF [2,5,45,46,47,48], bFGF [46,47,48], IL-8 [2,47,48] and MMP-9 [2,47]. For the purpose of a personalised therapeutic approach, PBCs represent an ideal autologous cell type, since their simple harvest and ample availability makes them easy to employ [2,5,19]. The provision of a standardized ex vivo hypoxic microenvironment for PBCs, that simulates the one normally found in an in vivo wound, could thus enable obtaining physiological growth factor mixtures, of naturally-occurring protein factor concentrations and ratios [5,19,44]. These growth factors can be delivered in the form of hypoxia preconditioned plasma (HPP) or hypoxia preconditioned serum (HPS), depending on whether peripheral blood is, respectively, anticoagulated or allowed to clot before conditioning (Figure 1B) [2,19,44].

By following the natural sequence of the wound healing stages, which progresses from haemostasis through to angiogenesis, it becomes readily apparent that the above described peripheral blood-derived secretomes, i.e., platelet-rich plasma (PRP) and hypoxia preconditioned products (HPP/HPS), not only differ in their method of preparation, but also correspond to, and essentially mirror distinct phases of the wound healing program (Figure 1C). Thus, while PRP contains a high concentration of platelet-derived factors by employing platelet concentration via blood centrifugation [29], something that also naturally happens during haemostasis through platelet aggregation within the fibrin clot, HPS and HPP comprise mostly protein factors that are newly produced by PBCs as a result of hypoxic exposure during blood conditioning [2,44]. A comparative evaluation of these two secretome categories, with respect to their angiogenic potential, is therefore a reasonable attempt towards understanding the relative contribution of these different cell types (platelets vs. PBCs) towards wound angiogenesis, as well as essential for gaining a greater insight into their specific clinical utility in wound healing and tissue repair therapies.

In the current study, we aimed to characterize these peripheral blood-derived secretomes, in terms of their key pro-angiogenic factor (VEGF) and anti-angiogenic factor (PF-4, TSP-1) composition, and analyse in vitro their ability to induce microvessel formation and sprouting in endothelial cell and aortic ring cultures, respectively. Furthermore, the effect of secretome freeze-storage on growth factor concentration and bioactivity, as well as the effect of secretome dilution on angiogenic potential were tested, parameters that are ultimately relevant for clinical application.

## 2. Materials and Methods

### 2.1. Preparation of Platelet-Derived Secretomes—Platelet Rich Plasma (PRP)

All blood donors provided written informed consent as directed by the ethics committee of the Technical University Munich, Germany, which approved this study (File Nr.: 497/16S). The preparation of PRP followed an established, double-centrifugation protocol [49] (see Figure 1A). In the first step, 6.5 mL venous blood was collected in blood tubes pre-filled with citrate anticoagulant (BD Vacutainer, Becton, Dickinson and Company, USA), under sterile and standardized conditions. Next, the blood was centrifuged at 160 × *g* for 20 min. After centrifugation, the blood was separated into three layers, from top to bottom: platelet-poor plasma (PPP), buffy coat (platelets and white blood cells) and red blood cells (RBCs). A mark was then placed 6 mm below the upper edge of the blood cell component (BCC), and all content above the mark (containing the buffy coat) was pipetted and transferred into a new sterile tube without anticoagulant, then centrifuged again for 15 min at 400× *g*. This second centrifugation resulted into separation of PRP and serum component (SEC). The PRP (approx. 0.5 mL) was separated from the SEC. The in vitro activation of PRP was carried out either with 0.5 mL of 1 I.U. Thrombin/mL and 1.7 mg/mL CaCl_2_ (Tisseel, Baxter, Germany) or with CaCl_2_ only in a concentration of 3.4 mg/mL. After an incubation period of 30 min at 37 °C, a third centrifugation (20 min, 160× *g*) was carried out. Then, 0.5 mL of the activated PRP supernatant was analyzed.

### 2.2. Preparation of Hypoxia Preconditioned Secretomes—Hypoxia Preconditioned Serum (HPS)/Plasma (HPP)

In the first step, 20 mL venous blood was collected in a 30 mL polypropylene syringe (Omnifix^®^, B Braun AG, Germany) that contained no additive or was pre-filled with 1 mL heparin (Medunasal^®^–Heparin 500 I.U. 5 mL ampoules), or in blood tubes pre-filled with EDTA anticoagulant (BD Vacutainer, Becton, Dickinson and Company, USA), under sterile and standardized conditions (Blood Collection Set; Safety-Lok, CE 0050, BD Vacutainer, Becton, Dickinson and Company, USA). Then, a 0.2 µm pore filter was attached (Sterifix^®^, CE 0123, Braun Melsungen AG, Germany) and by pulling the plunger, 5 mL air was drawn into the syringe. Subsequently, the filter was removed and the capped syringe was placed upright in an incubator (37 °C/5% CO_2_) and incubated for 4 or 7 days (blood incubation time), without any prior centrifugation. Pericellular local hypoxia (~1% O_2_) was induced in situ through cell-mediated O_2_ consumption, by controlling the blood volume per unit area (BVUA > 1 mL/cm^2^), and consequently the PBC seeding density in the blood container [13,19]. After the predefined incubation time, the blood was passively separated into three layers, from top to bottom; plasma/serum, clot/buffy coat, red blood cell component, so that the top layer comprising hypoxia preconditioned plasma or serum (HPP/HPS) could be filtered (0.2 µm pore filter, Sterifix^®^, B Braun AG, Germany) into a new syringe (see Figure 1B), removing cells/cellular debris. Through the step of filtration, the serum/plasma was rendered cell-free. HPP and HPS secretomes were then sampled, and partially diluted as required with phosphate buffered saline (PBS) at dilutions of 1:1; 1:2; 1:5; 1:10; 1:50; 1:100; 1:500; 1:1000, before being analyzed.

### 2.3. Preparation of Blood-Derived Secretomes

In the first step 20 mL venous blood was collected in a 30 mL polypropylene syringe (Omnifix^®^, B Braun AG, Germany) that contained no additive or was pre-filled with 1 mL heparin (Medunasal^®^– Heparin 500 I.U. 5 mL ampoules), or in blood tubes pre-filled with EDTA anticoagulant (BD Vacutainer, Becton, Dickinson and Company, USA), under sterile and standardized conditions (Blood Collection Set; Safety-Lok, CE 0050, BD Vacutainer, Becton, Dickinson and Company, USA). Blood with EDTA or heparin anticoagulant was centrifuged for 10 min at 160× *g* to obtain plasma samples. Plasma samples were also prepared with heparin anticoagulant and simple sedimentation for 60 min, without centrifugation. After sedimentation or centrifugation, the blood was separated into the known layers (see Section 2.1 and Section 2.2), so that the top layer (plasma or serum) could be filtered into a new syringe. Plasma or serum samples were collected and partially diluted with PBS at dilutions of 1:1; 1:2; 1:5; 1:10; 1:50; 1:100; 1:500; 1:1000, before being analyzed.

To test the anticoagulant EDTA for its angiogenic effect, 5 mL of PBS was added to the vacutainers containing EDTA and analyzed after sufficient mixing. Furthermore, the anticoagulant heparin and the PRP activators Thrombin and CaCl_2_ were tested at the previously mentioned concentrations (see Section 2.1). PBS medium and recombinant VEGF (90 ng/mL) were also tested as negative and positive controls, respectively.

### 2.4. Quantitative Analysis of VEGF, TSP-1 and PF-4 Concentration in Blood-Derived Secretomes

Blood-derived secretomes were sampled and analyzed by ELISA for VEGF, TSP-1 and PF-4 (R&D, USA), according to manufacturer’s instructions. Factor concentrations in blood-derived secretomes were measured immediately after the predefined incubation period (4 or 7 days), and after storage for 4 or 12 weeks at −20 °C degrees, as indicated. Five samples were tested per condition.

### 2.5. Analysis of the Effect of Blood-Derived Secretomes on In Vitro Microvessel Formation

The angiogenic potential of blood-derived secretomes was tested in an in vitro angiogenesis assay, by assessing their ability to induce tube formation in human umbilical vein endothelial cells (HUVECs, CellSystems, Germany), seeded on factor-reduced Matrigel (BD, Germany). HUVECS were seeded at a density of 10 × 10^3^ well, with 50 μL of test or control media added per well (μ-Slide Angiogenesis, Ibidi, Germany), and cultured in 5% CO_2_/37 °C for 12 h. Cells were then stained with Calcein AM (PromoKine, Germany), and tube formation was observed with fluorescence and phase contrast microscopy. Assessment of the extent of capillary-like network formation was carried out by counting the number of tubes and nodes (a node was defined as the point of intersection of two or more tubules), and quantification of tube length was carried out with image analysis using imageJ software (NIH, USA). Blood-derived secretomes were tested immediately after the predefined incubation period (4 or 7 days), and after storage for 4 or 12 weeks at −20 °C degrees, as indicated. At least three wells were tested per sample (*n* = 5), per condition.

### 2.6. Analysis of the Effect of Blood-Derived Secretomes on In Vitro Microvessel Sprouting

Blood-derived secretomes were tested in the aortic ring assay, to assess their ability to induce microvessel sprouting. Aortic rings were dissected from female adult mice as previously described [50], underwent overnight serum starvation in opti-MEM reduced serum medium (Life Technologies, Germany) and embedded into Matrigel bilayer matrix (50 μL/layer in 96-well plates) (BD, Germany). Test and control secretomes, and control media were added (150 μL/well) to the rings, before culturing them in 5% CO_2_/37 °C. Medium change was carried out every 3 days, while rings were observed with phase contrast microscopy at 0, 3, 6 and 8 days and photographed, with all 4 quarters per ring analyzed for sprouting (formation of structures of connected cells that were attached, at their base, to the ring). Furthermore, tube length was quantified after a culture period of 8 days with image analysis using imageJ software (NIH, USA). At least three aortic rings were tested per sample (*n* = 5).

### 2.7. Statistical Analysis

For each experimental condition, *n* = 5 subjects were tested (in certain cases *n* = 3 or 4 subjects were tested, as noted). Data are expressed as mean ± standard deviation. Statistical analysis was carried out using Student’s independent t-test where a maximum of two groups was compared or repeated-measures ANOVA with Bonferroni adjustment, accompanied by post-hoc pairwise comparisons for analysis of three or more groups, using SPSS 14 software. Mauchly’s test was used to assess violation of sphericity in repeated-measures ANOVA, and in instances where Mauchly’s test was significant, degrees of freedom were corrected using Greenhouse–Geisser estimates of sphericity. The probability of a type one error was set to 5% (α = 0.05), unless noted otherwise.

## 3. Results

### 3.1. Quantitative Analysis of Pro- (VEGF) and Anti-Angiogenic (TSP-1, PF-4) Growth Factor Concentration in Blood-Derived Secretomes

**Figure 2:**
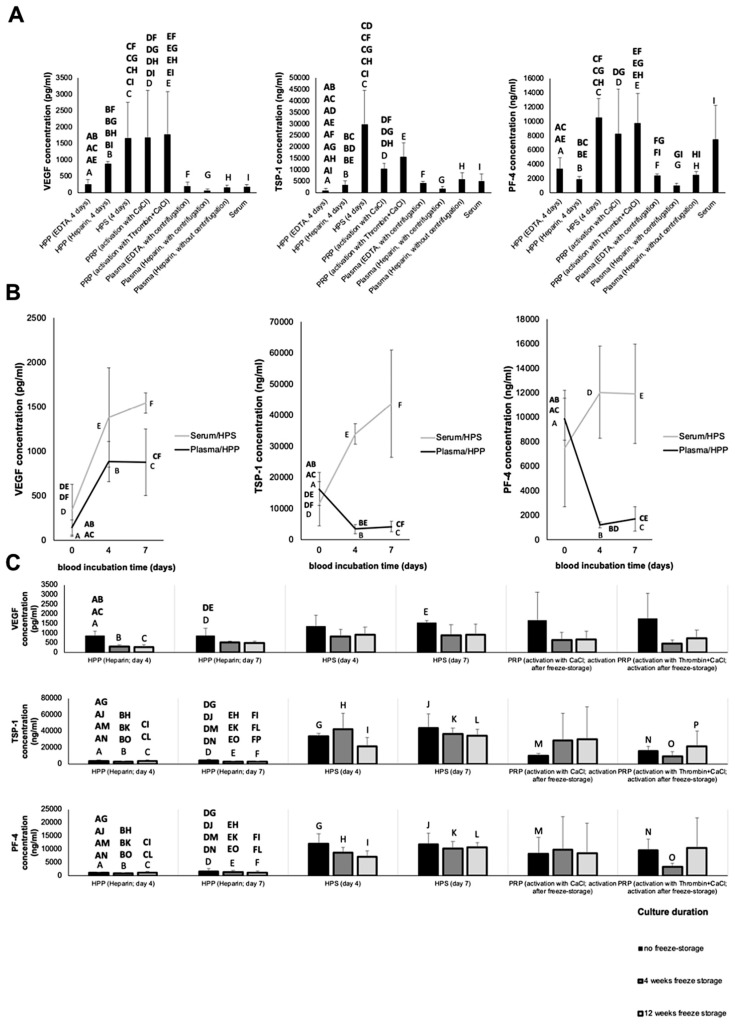
Quantitative analysis of pro- (VEGF) and anti-angiogenic (TSP-1, PF-4) factor concentration in blood-derived secretomes. (**A**) Plot showing the concentration of VEGF (pg/mL); TSP-1 (ng/mL) and PF-4 (ng/mL) in the various blood-derived secretomes tested (*n* = 5). Note that HPP and HPS were prepared without centrifugation. EDTA and heparin were used as blood anticoagulants in HPP and plasma preparation. (**B**) Effect of blood incubation time (days) on pro- (VEGF) and anti- (TSP-1, PF-4) angiogenic factor concentration in hypoxia preconditioned secretomes over 4 and 7 days preconditioning (*n* = 5). (**C**) Effect of freeze-storage on factor bioavailability. Plots comparing the VEGF, TSP-1 and PF-4 concentrations in fresh blood-derived secretomes and in secretomes stored for 4 and 12 weeks at −20 °C before testing (*n* = 5). Capital letter pairs over plots indicate statistical comparison of corresponding data points. For all pair comparisons, *p* < 0.05, unless otherwise indicated. Error bars represent s.d.

To establish a growth factor concentration baseline, we first quantitatively analyzed via ELISA the concentration of key angiogenesis-related protein factors (VEGF, TSP-1, PF-4) in normal plasma and serum, before proceeding to test their concentration in PRP and HPP/HPS. As shown in Figure 2A, the concentration of the pro-angiogenic factor VEGF in hypoxia preconditioned secretomes and platelet-derived secretomes showed a significant 3- to 5-fold increase compared to their baseline level in fresh plasma (*p* < 0.01) and fresh serum (*p* < 0.05). Due to a large standard deviation in factor levels, no statistically significant differences could be observed between the three tested secretomes, except for HPP prepared with EDTA as anticoagulant, which had a significantly lower VEGF concentration (*p* < 0.001). The concentration of the platelet-derived angiogenic inhibitors TSP-1 and PF-4 in both fresh plasma and HPP (prepared with either EDTA or heparin anticoagulant) was significantly lower than that in HPS and PRP (*p* < 0.01) (Figure 2A), indicating that the process of blood conditioning used for HPP preparation did not significantly increase platelet activation. Indeed, no significant difference in the concentration of any of the factors tested could be observed when plasma preparation (following heparin anticoagulation) was carried out through passive sedimentation of blood, as also used for HPP preparation, or conventionally through blood centrifugation (Figure 2A). The method of blood anticoagulation used for HPP preparation (EDTA vs. heparin) appeared to influence VEGF and TSP-1 levels (lower levels with EDTA than heparin anticoagulation), but not PF-4, suggesting that EDTA primarily influenced hypoxia-regulated factor expression, rather than platelet activation, in HPP. The type of PRP activation used (CaCl_2_ vs. thrombin + CaCl_2_) did not appear to exert any significant influence on the concentration of these three factors being released in PRP (Figure 2A).

Based on these findings, a more detailed characterization of hypoxia preconditioned secretomes was undertaken, in terms of the effect of varying the duration of blood incubation time on factor levels. Physiological hypoxia (1% O_2_) was generated in situ through cell-mediated O_2_ consumption, as previously described [13,19] (see Materials and Methods Section 2.2). The positive effect of hypoxia on VEGF upregulation was evident over the tested incubation period of 7 days (Figure 2B). Indeed, as it had been expected from the data of Figure 2A, a 3- to 4-fold increase in the VEGF concentration of HPP and HPS was observed following 4 and 7 days blood incubation, compared to baseline values in fresh plasma and serum (no blood incubation), respectively (*p* < 0.05) (Figure 2B). In direct comparison, the VEGF concentration of 7 days -incubated HPS was significantly greater than that in HPP (*p* < 0.01), although this difference was absent at 4 days incubation (Figure 2B). With respect to the anti-angiogenic factors, TSP-1 and PF-4 concentrations in 4 days -incubated HPP were more than threefold lower than those in fresh plasma, an effect persisting over 7 days of blood incubation (*p* < 0.01) (Figure 2B). In contrast, TSP-1 concentration in HPS was threefold higher compared to fresh serum after 4 and 7 days incubation (*p* < 0.01). No significant difference was seen in PF-4 levels between HPS (either incubation period) and fresh serum. Nonetheless, significant differences could be recorded between HPP and HPS with respect to both TSP-1 and PF-4 concentration, at 4 and 7 days incubation (*p* < 0.05) (Figure 2B).

To examine whether the tested blood-derived secretomes could maintain their proteomic bioavailability when stored at low temperature (−20 °C), what would admittedly be a clinically useful property for off-the shelf clinical application, HPP/HPS and PRP samples were frozen for 4 and 12 weeks following preparation, before factor protein levels were quantified via ELISA. As shown in Figure 2C, there was a significant drop in the HPP concentration of bioavailable VEGF in samples obtained after 4 days blood incubation (*p* < 0.01), but not 7 days, suggesting an initially more robust VEGF upregulation following 7 days blood preconditioning. Nonetheless, VEGF remained detectable in all frozen HPP samples tested, even after 12 weeks freeze-storage. Furthermore, neither 4 nor 12 weeks freeze-storage seemed to significantly affect VEGF bioavailability in HPS and PRP. Importantly, no differences could be seen in TSP-1 and PF-4 levels between fresh and frozen secretomes (HPP, HPS, PRP). This could be indirectly confirmed, through the maintenance of a significantly higher TSP-1 and PF-4 concentration in frozen HPS and PRP samples compared to frozen HPP samples (*p* < 0.05) (Figure 2C).

### 3.2. Analysis of the Ability of Blood-Derived Secretomes to Induce Microvessel Formation In Vitro

**Figure 3:**
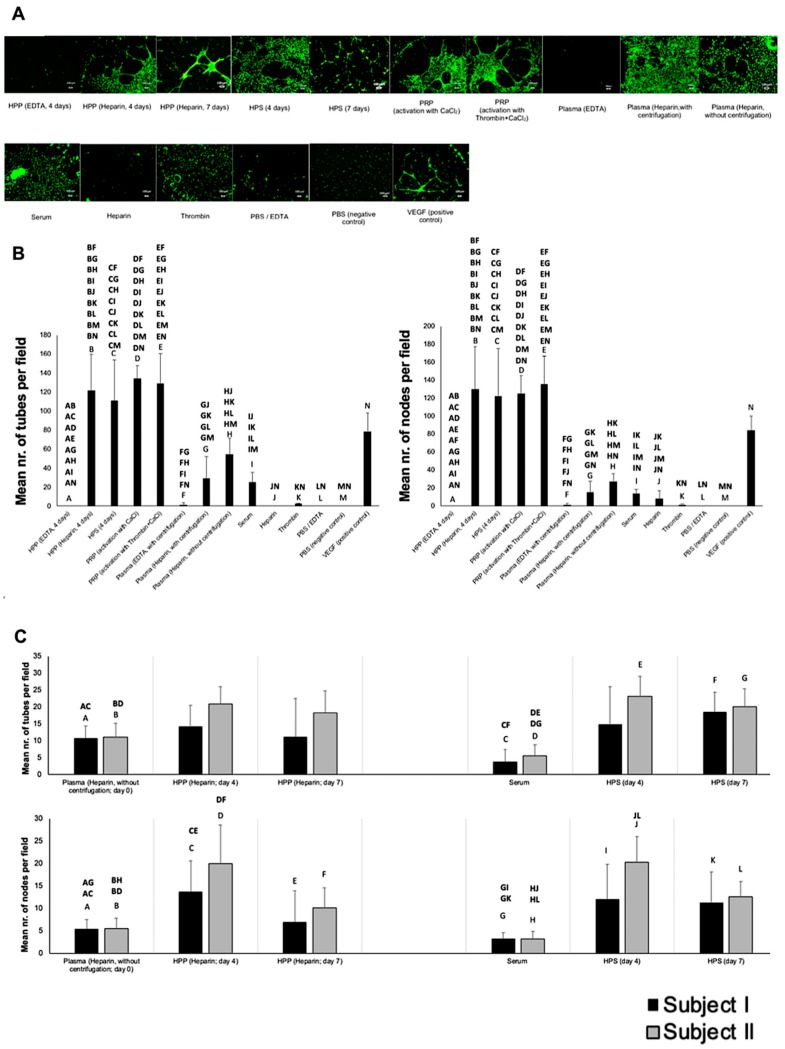
Effect of blood-derived secretomes on microvessel formation in human umbilical vein endothelial cell (HUVEC) cultures in vitro. (**A**) Panel showing representative images of the tube formation assay (12 h), carried out in the presence of the shown blood-derived secretomes (Bars = 100 µm). (**B**) Plot showing the mean number of tubes (left) and nodes (right) formed in different blood-derived secretome cultures (*n* = 5). (**C**) Effect of blood incubation time (4 and 7 days) on the angiogenic activity of hypoxia preconditioned secretomes. Plot showing the mean number of tubes (top) and nodes (bottom) formed in 4 and 7 days incubated -HPP and -HPS cultures, with blood obtained from two subjects (*n* = 3 per subject). Capital letter pairs over plots indicate statistical comparison of corresponding data points. For all pair comparisons *p* < 0.05, unless otherwise indicated. Error bars represent s.d.

Following an analysis of pro- and anti-angiogenic factor concentration in blood-derived secretomes, we moved on to investigate their ability to induce microvessel formation in human umbilical vein endothelial cell (HUVEC) in vitro cultures. We found a 3- to 4-fold increase in the number of tubes (*p* < 0.05), and a 4- to 5-fold increase in the number of nodes (*p* < 0.05) in hypoxia preconditioned and platelet-derived secretomes compared to baseline levels in fresh plasma and serum (Figure 3A,B). No significant difference in the induced angiogenic response was observed between hypoxia preconditioned and platelet-derived secretomes, except for HPP prepared with EDTA as anticoagulant, which showed minimal tube formation. In support of our ELISA results, the omission of centrifugation in plasma preparation, as well as the method used for PRP activation (CaCl_2_ vs. thrombin + CaCl_2_) did not significantly influence the degree of tube formation.

An assessment of microvessel formation in cultures with hypoxia preconditioned secretomes that were prepared with a varying blood incubation time (4 vs. 7 days) showed that preconditioning improved the angiogenic potential of serum, which was initially less angiogenic than fresh plasma (*p* < 0.001) (Figure 3A,C). In particular, HPS prepared with both 4 and 7 days blood incubation generated 3 to 4 times as many tubes (*p* < 0.001) and nodes (*p* < 0.05) as fresh serum, although no significant differences were observed between the two incubation periods. In HPP cultures, 4 days of blood preconditioning had a positive effect on the number of nodes formed compared to fresh plasma (*p* < 0.001), although no significant difference was found in terms of tube formation (Figure 3A,C).

**Figure 4:**
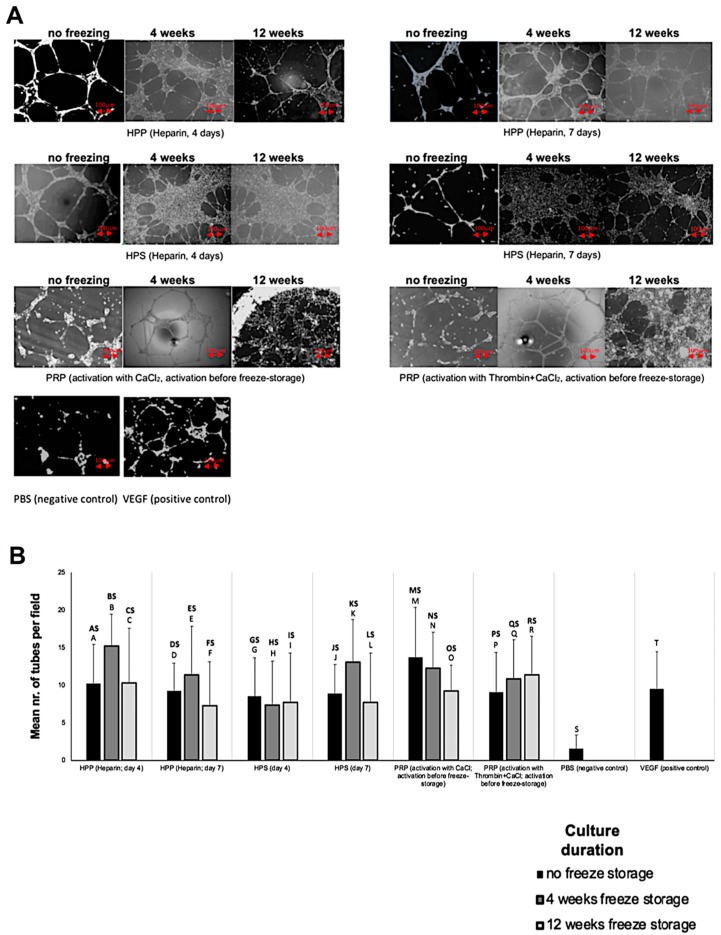
Effect of blood-derived secretome freeze-storage on angiogenic activity. (**A**) Panel showing representative images of the tube formation assay carried out with HPP, HPS and PRP secretomes that underwent freeze storage for 4 and 12 weeks (Bars = 100 µm). (**B**) Plot comparing the mean number of tubes formed in endothelial cell cultures in the presence of fresh blood-derived secretomes or secretomes that underwent freeze-storage for 4 and 12 weeks at −20 °C (*n* = 5) (*p* < 0.001). Capital letter pairs over plots indicate statistical comparison of corresponding data points. For all pair comparisons *p* < 0.05, unless otherwise indicated. Error bars represent s.d.

We also tested the functional bioactivity of freeze-stored secretomes in the in vitro angiogenesis assay. As expected from our VEGF data (see Figure 2C), freezing hypoxia preconditioned and platelet-derived secretomes did not appear to impair their angiogenicity, with all secretomes inducing significantly greater tube formation than phosphate buffered saline (PBS), used here as negative control (*p* < 0.001) (Figure 4A,B). Moreover, no difference in bioactivity was detected between secretomes, for any of the two freeze-storage periods tested.

### 3.3. Analysis of the Ability of Blood-Derived Secretomes to Induce Microvessel Sprouting In Vitro

**Figure 5:**
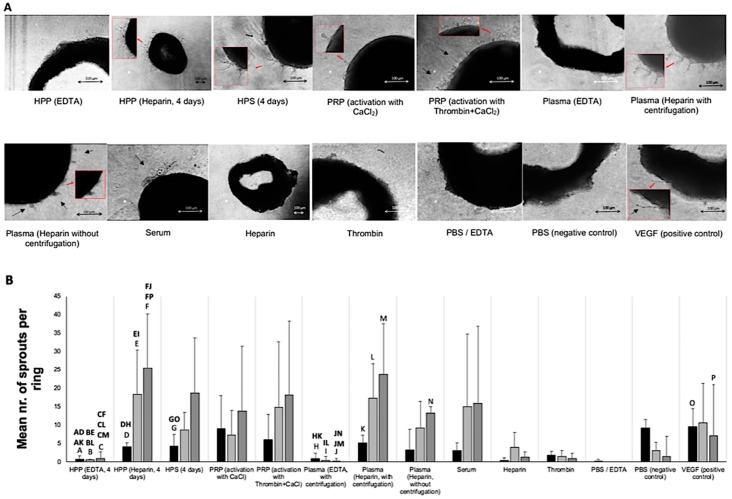

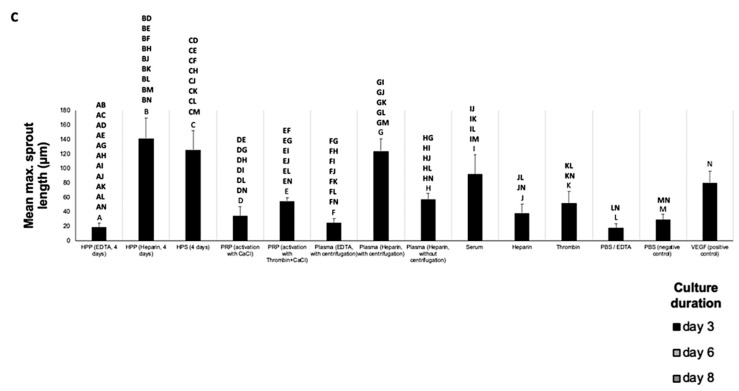
Effect of blood-derived secretomes on microvessel sprouting in the aortic ring assay in vitro. (**A**) Panel showing representative images of aortic rings, embedded in Matrigel and cultured in the presence of the shown blood-derived secretomes for 3, 6 and 8 days. (Bars = 100 µm). Microvessels sprouting from aortic rings are indicated by black arrows. Enlarged image sections are indicated by red insets. (**B**) Plot showing the mean number of sprouts per ring over a culture duration of 3, 6 and 8 days (*n* = 3). (**C**) Plot showing the mean maximum sprout length after a culture period of 8 days (*n* = 3). Capital letter pairs over plots indicate statistical comparison of corresponding data points. For all pair comparisons, *p* < 0.05, unless otherwise indicated. Error bars represent s.d.

Having assessed the ability of the various blood-derived secretomes to induce microvessel formation in vitro, an analysis of microvessel sprouting was carried out using the mouse aortic ring assay. Screening for sprouting angiogenesis only showed small differences between hypoxia preconditioned and platelet-derived secretomes (Figure 5A,B). In all secretome cultures, we observed a trend for an increasing number of sprouts as culture duration increased (3, 6 and 8 days), although such differences were again non-significant, due to the high standard deviation seen between samples (Figure 5B). Similar to the tube formation assay, fresh plasma and HPP prepared with EDTA as anticoagulant induced significantly a smaller number of sprouts than their heparin-anticoagulated counterparts (*p* < 0.001).

In contrast to the sprout number, hypoxia preconditioned secretomes (heparin- anticoagulated HPP, and HPS) generated a 2- to 3-fold greater mean sprout length than platelet-derived secretomes (*p* < 0.01) after a culture period of 8 days, when the longest microvessels could be observed (Figure 5C). Moreover, heparin-anticoagulated HPP induced significantly longer sprouts than pure VEGF, used here as positive control (*p* < 0.05). In agreement with our analysis of sprout number, sprout length was found to be negatively impacted when EDTA was used as anticoagulant in fresh plasma and HPP (Figure 5C). Fresh serum generated equally long sprouts as HPS, but significantly longer sprouts than PRP (*p* < 0.05), indicating that while both hypoxia-induced and platelet-derived factors seemed to be important for sprout lengthening, an oversupply of platelet-derived factors appeared to limit sprout extension.

### 3.4. Effect of Hypoxia Preconditioned Secretome Dilution on Angiogenic Activity

**Figure 6:**
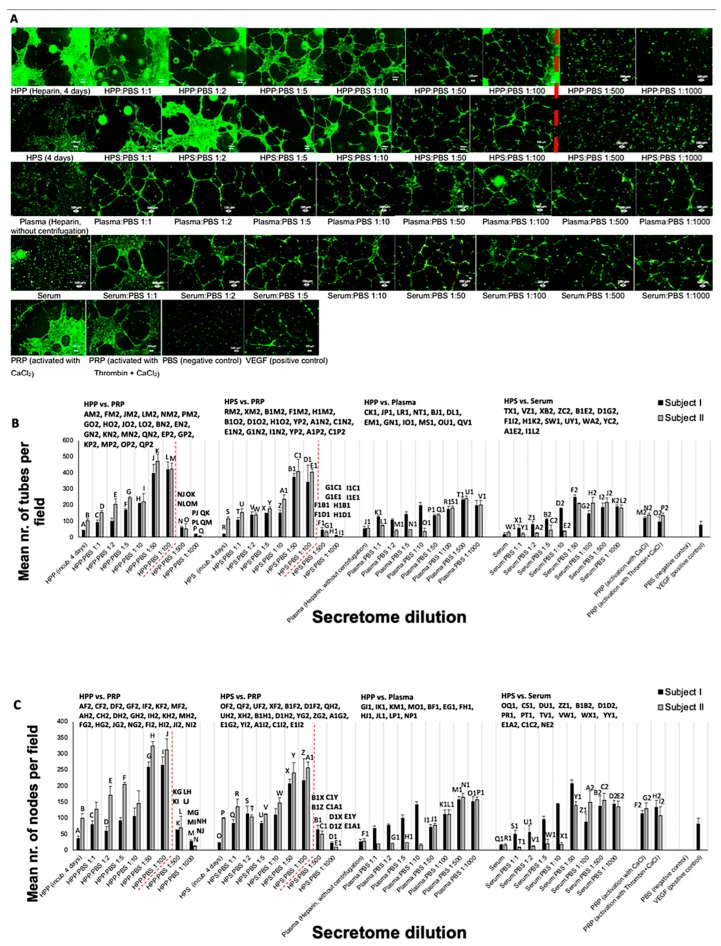
Effect of hypoxia preconditioned secretome dilution on microvessel formation in HUVEC cultures in vitro. (**A**) Panel showing representative images of the tube formation assay (12 h), carried out in the presence of the shown diluted blood-derived secretomes (Bars = 100 µm). (**B**,**C**) Plot showing the mean number of tubes (**B**) and nodes (**C**) formed in cultures with different blood-derived secretome and basal control dilutions (1:1 to 1:1000), for blood obtained from two individual subjects (*n* = 3 per subject). The red dashed line indicates the statistically significant cut-off point of dilution. Capital letter pairs shown directly above histograms indicate intra-condition statistical comparison of corresponding data points, while HPP/HPS vs. Plasma/Serum and HPP/HPS vs. PRP statistical comparisons are shown on the upper part of plot B and C. For all pair comparisons *p* < 0.05, unless otherwise indicated. Error bars represent s.d.

With the assumption that following in vivo secretome delivery, growth factor release and diffusion into the surrounding microenvironment would evidently result in some degree of reduction in the local protein factor concentration, we moved on to test the angiogenic potential of increasing dilutions (1:1 to 1:1000) of hypoxia preconditioned secretomes and basal plasma/serum controls, as this could be informative towards potentially identifying clinically effective dosage. In this experiment, we deliberately avoided diluting PRP, as this is per definition a concentrated secretome (i.e., diluted PRP would resemble normal plasma). Increasing the dilution of HPP and HPS up to 1:100 produced a progressively stronger angiogenic response in terms of the number of tubes and nodes formed in endothelial cell cultures (Figure 6A–C). Beyond this dilution level, however, the angiogenic response diminished rapidly (*p* < 0.01). The distinct bell-shaped profile observed in HPP/HPS was not clearly evident within the range of dilutions tested for fresh plasma and serum, implying a delay phenomenon in the dilution effect, as confirmed by the significantly greater response induced by 1:100 diluted HPP and HPS compared to 1:100 diluted plasma (*p* < 0.001) and serum (*p* < 0.05), respectively (Figure 6B,C). HPP and HPS with a 1:2 dilution demonstrated similar mean number of tubes and nodes as platelet-derived secretomes. Significant differences could be revealed, however, in the range of 1:5–1:100 dilution, with HPP/HPS surpassing the PRP angiogenic response (*p* < 0.01) (Figure 6B,C). Higher HPP/HPS dilutions (1:500 and 1:1000), on the other hand, generated less tubes and nodes than PRP (*p* < 0.001) (Figure 6B,C). Importantly, no significant difference could be observed between the angiogenic response induced by PRP and fresh plasma or serum diluted by 1:50 to 1:1000 (Figure 6B,C).

**Figure 7:**
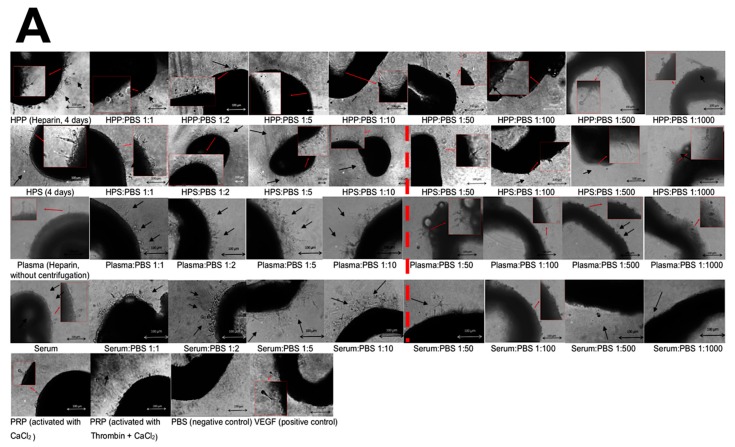

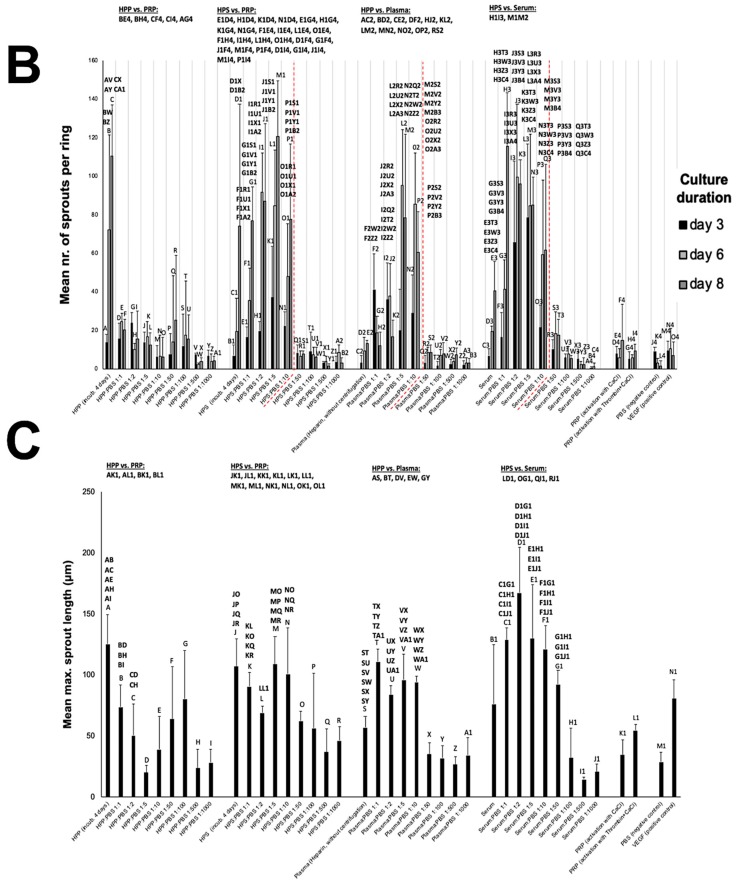
Effect of hypoxia preconditioned secretome dilution on microvessel sprouting in vitro. (**A**) Panel showing representative images of aortic rings, embedded in Matrigel and cultured in the presence of diluted blood-derived secretomes for 3, 6 and 8 days (Bars = 100 µm). Microvessels sprouting from aortic rings are indicated by black arrows. Enlarged image sections are shown by red insets. (**B**) Plot showing the mean number of sprouts per ring over a culture duration of 3,6 and 8 days (*n* = 3). (**C**) Plot showing the mean maximum sprout length after a culture period of 8 days (*n* = 3). Red dashed lines on plots and images indicate statistically significant cut-off points of dilution. Capital letter pairs shown directly above histograms indicate intra-condition statistical comparison of corresponding data points, while HPP/HPS vs. Plasma/Serum and HPP/HPS vs. PRP statistical comparisons are shown on the upper part of plot B and C. For all pair comparisons *p* < 0.05, unless otherwise indicated. Error bars represent s.d.

The effect of secretome dilution was also tested in the aortic ring assay, which showed that microvessel sprouting increased with a more prolonged culture time (3, 6 and 8 days), and behaved in a similar manner to tube formation, with increasing dilutions generating a gradually greater response, although here differences were less significant due to the high standard deviation seen between samples (Figure 7A,B). HPS, as well as fresh serum and plasma produced the previously seen bell-shaped profile, but with the cut-off point being observed earlier, at the 1:10 dilution (Figure 7A,B). No distinct peak was observed in the HPP dilution series, although here dilutions equal to or greater than 1:500 also generated only a very small response. According to our previous data, HPS and PRP generated a similar mean number of sprouts, however, HPS dilutions of 1:1 to 1:10 generally produced a larger number of sprouts than PRP (*p* < 0.05) (Figure 7A,B). In addition, fresh plasma dilutions of 1:1 to 1:10 performed better in terms of sprouting than PRP (*p* < 0.05). With respect to the length of sprouts, no clear bell-shaped profile could be observed in any of the tested secretomes (Figure 7C). HPP and HPS generated significantly longer sprouts than PRP, up to dilutions of 1:1 and 1:50, respectively (*p* < 0.01). PRP also appeared to underperform diluted fresh plasma and serum, up to dilutions of 1:10 (*p* < 0.05).

## 4. Discussion

Peripheral blood has been, for considerable time, thought of as a suitable source of growth factor proteins that can be used to aid tissue repair and regeneration [2,5,8,44,51,52]. This is indeed a sound rational, given the interwoven participation of peripheral blood cells in all stages of the wound healing process [6,8,53,54]. Among the various blood-based products that have been clinically tested, platelet-derived secretomes such as platelet-rich plasma (PRP) and platelet-rich fibrin matrix (PRFM) have received by far the greatest attention [3,4,55]. Hypoxia preconditioned blood-derived secretomes (HPP and HPS) represent a newer development in the field of autologous growth factor preparations, and offer a novel alternative to platelet-derived secretomes [2,5,19,44]. The basic idea underlying the proposed functionality of all these products is the ex vivo recapitulation of the physiological cellular responses involved in wound repair, by employing protein factor signalling that is naturally emitted by key cell types, namely platelets in the PRP approach, and peripheral blood cells (PBCs: neutrophils, macrophages, lymphocytes) in HPP/HPS (see Figure 1C). In contrast to simple blood centrifugation and subsequent concentration of cells (platelets), and their growth factors, blood preconditioning under physiological temperature and hypoxia (i.e., extracorporeal wound simulation) offers a means of optimizing the angiogenic potential of blood-based products through hypoxia-induced temporal changes in PBC growth factor expression, without merely relying on the release of factors already stored within platelets at the time of blood collection [2,5,19,44]. Furthermore, by allowing PBCs to regulate their O_2_ microenvironment, as employed in this work, instead of exposing them to an artificial one (i.e., fixed/global hypoxia produced within an O_2_ controlling incubator), it may even be possible to better simulate the conditions encountered within an in vivo wound [16,17,18,19], since the direct correlation of pericellular O_2_ tension with hypoxia-regulated gene expression [5,13] suggests that a more physiological angiogenic response could be achieved through cell-mediated hypoxia. Importantly, beyond the obvious differences in cell type employed (i.e., platelets vs. PBCs) and method of preparation used (i.e., centrifugation vs. hypoxic preconditioning), PRP and HPP/HPS organically differ with respect to their specific correlation with the wound healing phases, the former having a direct correlation with the haemostatic phase, while the latter being more closely correlated with the angiogenesis-driven proliferative phase (see Figure 1C). It could thus be argued that the development of hypoxia preconditioned secretomes represents a true paradigm shift in the evolution of blood-based products, since it offers a logical progression along the wound healing pathway itself, pushing the focus away from the initial trauma-induced signalling phase, towards the more specific hypoxia-driven angiogenic response.

In this work we hypothesized that the aforementioned fundamental differences in the preparation of these secretomes would translate into measurable differences in the concentration of key pro- (VEGF) and anti- (TSP-1, PF-4) angiogenic factor proteins. We deliberately chose to focus on hypoxia-regulated factors (VEGF, TSP-1) and platelet-derived factors (VEGF, TPS-1, PF-4), in order to identify whether the method of secretome preparation, i.e., hypoxic preconditioning vs platelet concentration, influenced their angiogenic composition. We found that both PRP and HPP/HPS had a significantly higher VEGF concentration compared to their baseline controls, i.e., fresh plasma and fresh serum (see Figure 2A), while the levels of the platelet-derived factors TSP-1 and PF-4 in HPP were significantly lower than those in HPS and PRP, indicating minimal platelet activation during blood conditioning in HPP (see Figure 2A). Indeed, the method of blood anticoagulation used for HPP preparation, i.e., EDTA vs. heparin, appeared to only influence the levels of the hypoxia-regulated factors VEGF and TSP-1 (EDTA likely interfered with hypoxia-inducible factor (HIF) signalling [56]), but not PF-4, which is solely derived from platelets. The increased concentration of VEGF in HPP, which must therefore be primarily of non-platelet origin, thus supports the positive influence of hypoxic blood conditioning on PBC pro-angiogenic factor expression [2,5]. It should, of course, not be forgotten that the three factors tested here, while important in angiogenesis, only represent a tiny portion of the plethora of pro- and anti-angiogenic factor proteins that are known to exist in blood-derived secretomes [2,13,17,57], therefore, the differences between the growth factor profiles of these secretomes might be vastly greater than what can be inferred from our current results. For example, differences in their content and composition of matrix metalloproteinases (MMPs), which are known to be released by platelets and leukocytes, could significantly affect their utility in preventing/treating scar formation in traumatic and chronic wounds, with significant clinical implications Previous work from our group had employed mass spectrometry to analyse the proteomic composition of peripheral blood cell-derived secretomes in an in vitro wound model utilizing hypoxic stress stimulation [13]. Further proteome profiling using similar approaches might indeed highlight a greater degree of dissimilarity in their growth factor composition, and by extension in their in vivo functionality.

The statistically significant correlation of HPP/HPS VEGF concentration with an increasing blood incubation time of 4 to 7 days (see Figure 2B) confirmed our previous findings of hypoxia-induced pro-angiogenic factor upregulation in hypoxia preconditioned secretomes [2,5]. The higher VEGF concentration measured in 7 days-incubated HPS, compared to that measured in HPP, likely represents the additional platelet-derived VEGF that had been released into HPS following blood coagulation. In these experiments, HPP not only had a lower concentration of VEGF than HPS, but also a lower concentration of the platelet-derived angiogenesis inhibitors TSP-1 and PF-4, in agreement with the data of Figure 2A. The finding that TSP-1 and PF-4 concentrations in 4 and 7 days-incubated HPP were significantly lower than those in fresh plasma (see Figure 2B) might be explained by a net protein breakdown occurring during blood incubation, in the absence of continuous factor release from platelets. In contrast, hypoxia-induced upregulation of TSP-1 expression in HPS [2] resulted in a higher TSP-1 concentration in 4 and 7 days-incubated HPS than that in fresh serum, a difference not seen in the levels of PF-4 (see Figure 2B). Therefore, while HPP appears to comprise primarily hypoxia-induced pro-angiogenic signalling, and can be thus considered an angiogenesis-targeting growth factor mixture, HPS represents a more ‘complete’ secretome, since it provides both the coagulation-generated, as well as the hypoxia-induced phase of the wound healing cascade (see Figure 1C). Despite their differences, both HPP and HPS are characterized by the physiological optimization of their growth factor composition, which is achieved through hypoxia, rather than an artificial fortification in the concentration of platelet-derived factors, as in PRP. This, by definition, makes HPP and HPS more physiological secretomes than platelet-based products, with significant implications towards their functionality as promoters of tissue-regeneration.

An analysis of the ability of these blood-derived secretomes to induce microvessel formation in endothelial cell cultures in vitro revealed that both hypoxia preconditioned and platelet-derived secretomes were more potent than their corresponding baseline controls (fresh plasma and serum), with no significant difference in either the number of tubes or nodes being observed between the two secretome types (see Figure 3A,B). The latter is important, given the previously discussed differences in the concentration of anti-angiogenic factors between HPP and HPS/PRP, and highlights the dependence of new vessel formation on the presence of an adequate amount of pro-angiogenic factors, at least at the in vitro level. In accordance to our ELISA results (see Figure 2A), HPP prepared with EDTA as anticoagulant showed minimal tube formation, likely due its low concentration of pro-angiogenic factors (e.g., VEGF). The positive effect of hypoxia on secretome angiogenic potential could be confirmed by the finding that hypoxic preconditioning improved the angiogenic activity of serum (4 and 7 days-incubated HPS), which was initially less angiogenic than fresh plasma (see Figure 3A,C), an observation that is consistent with the fact that serum has a higher concentration of anti-angiogenic factors (e.g., TSP-1, PF-4). The length of blood conditioning (4 vs. 7 days) did not appear to significantly influence the angiogenic potency of HPP or HPS (see Figure 3A,C), in agreement with the plateau seen in VEGF concentration (see Figure 2B).

Assessment of the functional bioactivity of freeze-stored secretomes in the in vitro angiogenesis assay provided evidence that low-temperature storage did not negatively impact their ability to promote microvessel formation (see Figure 4A,B), which was in agreement with our ELISA data showing preservation of VEGF protein in all secretomes, following 4 and 12 weeks freeze storage (see Figure 2C). As in the case of fresh secretomes, no difference in angiogenic potency was detected between freeze-stored hypoxia preconditioned and platelet-derived secretomes (see Figure 4A,B). We note, however, that only the platelet-derived secretomes that had been activated in vitro *before* freezing, and were thus rendered cell-free, could be tested in this assay, since freezing caused cell haemolysis and prevented evaluation of PRP that had been activated after freeze-storage. This problem of platelet haemolysis might, however, present more than just a technical hurdle, since the presence of cellular debris in the final product might prohibit the freezing of PRP immediately after its preparation, in the clinical setting.

With respect to microvessel sprouting, no significant differences could be detected between hypoxia preconditioned and platelet-derived secretomes, while neither secretome type appeared to induce more sprouting in aortic ring cultures than fresh plasma or serum (see Figure 5A,B). Hypoxia preconditioned secretomes did, however, promote the formation of longer sprouts than PRP (see Figure 5C). This finding, together with the fact that fresh serum generated equally long sprouts as HPS, and significantly longer sprouts than PRP (see Figure 5C), seems to suggest that while both hypoxia-induced and platelet-derived factors are important for sprout lengthening, as previously suggested [58], an oversupply of platelet-derived factors, more specifically angiogenic inhibitors (e.g., PF-4), appears to limit sprout extension. Previous work from our group, and others, has demonstrated that microvessel formation and sprouting are two distinct cellular responses, which appear to rely on different sets of growth factor signalling rules [13,59]. In particular, while microvessel formation is strongly inhibited by the platelet-derived angiostatic factor PF-4, this factor seems to play an important role in sprouting angiogenesis [13,60]. Our new data indicate that while an abundance of PF-4, even at the supraphysiological concentrations found in PRP, does not limit microvessel sprouting, it might have a negative impact on the average extension of new sprouts. Further work is, however, required to fully clarify these effects, before a precise factor concentration range that optimally supports these responses can be defined.

**Figure 8:**
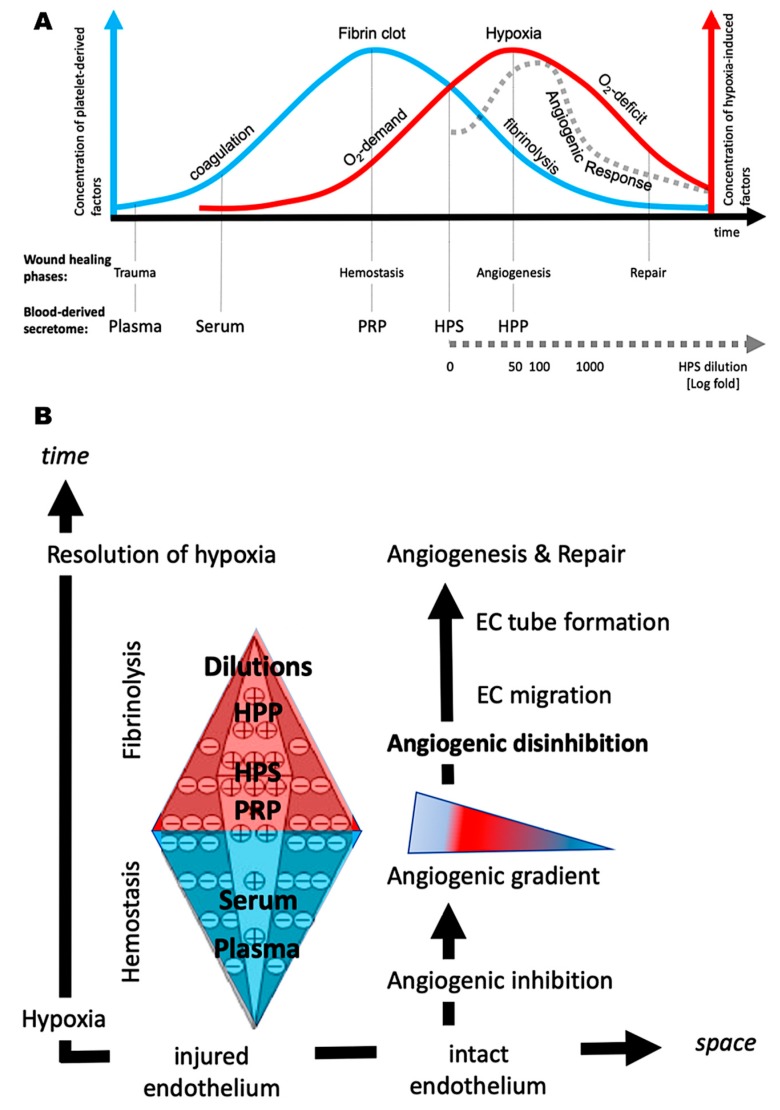
Biological basis underlying the utilization of different blood-derived secretomes. (**A**) Plot showing the representative correlation of different types of blood-derived secretomes (plasma, serum, PRP, HPS, HPP) with the progressive phases of wound repair. The coagulation-induced signaling phase (platelet-derived growth factors; blue curve) that supports haemostasis, correlates with PRP since it depends on the maximum concentration of platelets, while the hypoxia-induced signaling phase (PBC-derived growth factors; red curve) that drives angiogenesis, correlates initially with HPS, and eventually with HPP and HPS dilutions. The shape of the dotted gray curve represents the net angiogenic response, and is theoretically defined by subtracting anti-angiogenic platelet-derived signaling (blue curve) from pro-angiogenic hypoxia-induced signaling (red curve). (**B**) Proposed mechanism for the biochemical control of wound angiogenesis, and correlation of the various blood-derived secretomes (plasma, serum, PRP, HPS, HPP) with the different wound healing phases. Following completion of the haemostatic phase, the fibrin matrix undergoes controlled degradation through fibrinolysis, leading to depletion in the local pool of anti-angiogenic factors (e.g., PF-4) which are bound to fibrin (indicated by ‘’-‘’ sign), while up-regulation of pro-angiogenic factors (e.g., VEGF) though hypoxia (indicated by ‘’+’’ sign) leads to the formation of chemoattractive gradients that direct endothelial cell (EC) migration towards the injured site. Angiogenic disinhibition enables vascularization of the matrix through fibrinolysis-mediated EC invasion, which facilitates resolution of hypoxia and tissue repair.

Interestingly, diluting hypoxia preconditioned secretomes appeared to *increase* their ability to promote microvessel formation in the in vitro angiogenesis assay, rather than decrease it. This effect persisted up to a 100-fold dilution, beyond which a reduced angiogenic response was observed (see Figure 6). Furthermore, both HPP and HPS of 1:5 to 1:100 dilution were more potent than PRP, which is by its nature a concentrated product, and is known to contain large amounts of angiogenesis-inhibiting growth factors (e.g., PF4, TSP-1) [11,29,61]. Similar effects were observed in the sprouting assay, although here the maximum HPP/HPS dilution that could support sprouting was lower, at 1:10 (see Figure 7). Importantly, PRP was not found to be more angiogenic (microvessel formation and sprouting) than analogous dilutions of fresh plasma or serum (see Figure 6 and Figure 7), which casts doubt on the actual utility of concentrating platelet-derived secretomes as a power-tool for stimulating angiogenesis in vivo. When taken together into consideration, these findings suggest that physiological microvessel formation and sprouting are dependent on the fine balance of pro- and anti-angiogenic factors, rather than an oversupply of any single factor. As an oversimplification, the most favourable growth factor composition seems to be one that allows a relative reduction of angiogenesis-inhibitory factors, while maintaining an adequate amount of pro- angiogenic factors. Predictably, and as shown by our data, when the latter becomes too low, the response sharply declines. This mechanism would be in support of angiogenic disinhibition, instead of direct pro-angiogenic stimulation, as the primary switch that physiologically triggers hypoxia-induced angiogenesis in vivo (Figure 8A,B), and it is in agreement with the findings of our previous work which employed PF-4 blocking experiments in an in vitro wound healing model [13]. The proposed two-step optimization of PBC-derived secretomes, i.e., first through hypoxic stimulation, and subsequent controlled dilution, mirrors in fact the spatiotemporal evolution of growth factor signalling that powers the angiogenesis-driven proliferative phase of wound healing, and forms the foundation of tissue repair following haemostasis (Figure 8A,B).

From a cell-biological perspective, hypoxic preconditioning of peripheral blood may offer an additional advantage, beyond the direct hypoxia-induced upregulation and optimization of angiogenic growth factor signaling that we have presented and discussed here. Previous work has indeed demonstrated that hypoxic preconditioning increases the survival and angiogenic potency of peripheral blood mononuclear cells via oxidative stress resistance and reduced reactive oxygen species (ROS) accumulation, compared to normoxic culture [62]. Powered with that knowledge, it might be possible in the future to develop more advanced therapeutic strategies around the HPP/HPS concept, by combining peripheral blood hypoxic preconditioning together with hypoxia/reoxygenation (H/R) protocols, since it is known that H/R (but not hypoxia alone) enhances the activation of ROS-dependent intracellular signaling and as a result accelerates the rate of neovascularization in vivo [63]. The combination of hypoxia-induced pro-angiogenic growth factor signaling with ROS generation could potentially offer a more physiological, and by extension robust angiogenic response than either approach in isolation.

## 5. Conclusions

The findings of this study highlight the fact that the angiogenic potential of blood-derived secretomes is defined by the complex stoichiometry of their component pro- and anti-angiogenic factor proteins, rather than the concentration of one or more growth factors. This understanding, then establishes a basis that, at its core, strongly opposes the rational of concentrating cells (e.g., platelets) and their stored growth factors, as a means of optimizing the composition of a blood-derived product. In contrast to the PRP approach, hypoxic conditioning of peripheral blood generates compositions that are characterized by the physiological upregulation of hypoxia-induced pro-angiogenic signalling, and which can further be fine-tuned through controlled secretome dilution. By employing this method, complex compositions can be obtained that are relatively rich in pro-angiogenic growth factors (e.g., VEGF), but have a lower concentration of anti-angiogenic factors (e.g., PF-4), compared to PRP. Our data indicate that such optimized secretomes promote a stronger angiogenic response than PRP in vitro, and therefore merit further clinical investigation as useful tools for promoting tissue repair and regeneration. The ability to administer hypoxia preconditioned products as cell-free preparations further differentiates these from platelet-based products, since it makes them safe and suitable for off-the-shelf application via freeze-storage.

## Abbreviations

PBC	peripheral blood cells
HPS	hypoxia preconditioned serum
HPP	hypoxia preconditioned plasma
PRP	platelet-rich plasma
PRFM	platelet-rich fibrin matrix
VEGF	vascular endothelial growth factor
TSP-1	thrombospondin-1
PF-4	platelet factor-4
IL-8	interleukin-8
FGF	fibroblast growth factor
MMP-9	matrix metalloproteinase-9
PBS	phosphate buffered saline
